# The Role of Microbiota-Derived Metabolites in Colorectal Cancer

**DOI:** 10.3390/ijms24098024

**Published:** 2023-04-28

**Authors:** Coco Duizer, Marcel R. de Zoete

**Affiliations:** Department of Medical Microbiology, University Medical Center Utrecht, 3584 CX Utrecht, The Netherlands

**Keywords:** microbiota, metabolites, colorectal cancer, secondary bile acids, TMAO, SCFA, hydrogen sulfide, polyamines, indole, ADP-heptose

## Abstract

The impact of bacterial members of the microbiota on the development of colorectal cancer (CRC) has become clear in recent years. However, exactly how bacteria contribute to the development of cancer is often still up for debate. The impact of bacteria-derived metabolites, which can influence the development of CRC either in a promoting or inhibiting manner, is undeniable. Here, we discuss the effects of the most well-studied bacteria-derived metabolites associated with CRC, including secondary bile acids, short-chain fatty acids, trimethylamine-N-oxide and indoles. We show that the effects of individual metabolites on CRC development are often nuanced and dose- and location-dependent. In the coming years, the array of metabolites involved in CRC development will undoubtedly increase further, which will emphasize the need to focus on causation and mechanisms and the clearly defined roles of bacterial species within the microbiota.

## 1. Introduction

Colorectal cancer (CRC) is the third most prevalent cancer worldwide, accounting for approximately 10% of all cancer diagnoses and cancer-related deaths [1]. Currently, CRC is more prevalent in western countries, but cases are also rising in developing countries. The large population screening of people over 50 years old has contributed to the stabilization of CRC prevalence in that age group, while CRC prevalence in patients younger than 50 years old continues to rise [1].

CRC can be heritable, which is estimated to be the case in 12–35% of patients [1]. CRC can be characterized by mutagenic signatures, which mainly consist of one of two genome instabilities: chromosomal instability (CIN) or microsatellite instability (MSI) [2,3]. Alternatively, CRC can be classified as CpG island methylator phenotype (CIMP), which is characterized by hypermethylation across the genome [2,3]. Lastly, around 10% of CRC is CIN-, MSI- and CIMP-negative [2].

Chronic intestinal inflammation is also associated with the development of CRC and occurs in up to 5% of all tumors [3]. This is further exemplified in inflammatory bowel disease (IBD), in which patients are 2–3 times more likely to develop CRC in their lifetime compared to the general population [4]. It is believed that sustained intestinal inflammation induces oxidative damage and, subsequently, DNA damage, which in turn can drive CRC development [4]. It is important to note that IBD patients with CRC have the same genomic characteristics as other CRC patients [4]. 

There has been recent interest in the role of the intestinal microbiota in both IBD and CRC pathogenesis. In CRC, the most established bacterial contributors are specific strains of *Bacteroides fragilis*, *Escherichia coli*, *Fusobacterium nucleatum*, *Enterococcus faecalis*, *Peptostreptococcus anaerobius* and *Streptococcus anaerobius* [5,6]. While this list is non-exhaustive, how these species can mechanistically drive the development of CRC has been (partially) unraveled [5]. For the most part, research on the role of CRC-driving bacteria within the microbiota has focused on specific bacterial proteins (e.g., adhesins, as seen in *F. nucleatum*, or toxins, as seen in *B. fragilis*) [7,8,9,10]. However, there has recently been increased interest in the role of microbiota-derived metabolites that can stimulate CRC development. As the definition of ‘metabolite’ can be debatable, we define them as any molecule involved in bacterial metabolism, which are usually small (<1000 Dalton). Here, we review the known literature on the most influential and well-studied bacterial metabolites related to CRC pathogenesis.

## 2. Secondary Bile Acids

In order to allow the uptake of fat-soluble molecules and lipids, the liver produces primary bile acids that are excreted via the gallbladder into the duodenum [10,11,12,13]. These primary bile acids consist of cholic acid (CA) and chenodeoxycholic acid (CDCA) and are typically conjugated to glycine or taurine [10,11,12,13]. Alternatively, primary bile acids can be sulfated, which increases their solubility in water and results in more efficient excretion [14].

### 2.1. Conversion of Primary Bile Acids by the Intestinal Microbiota

**Figure 1:**
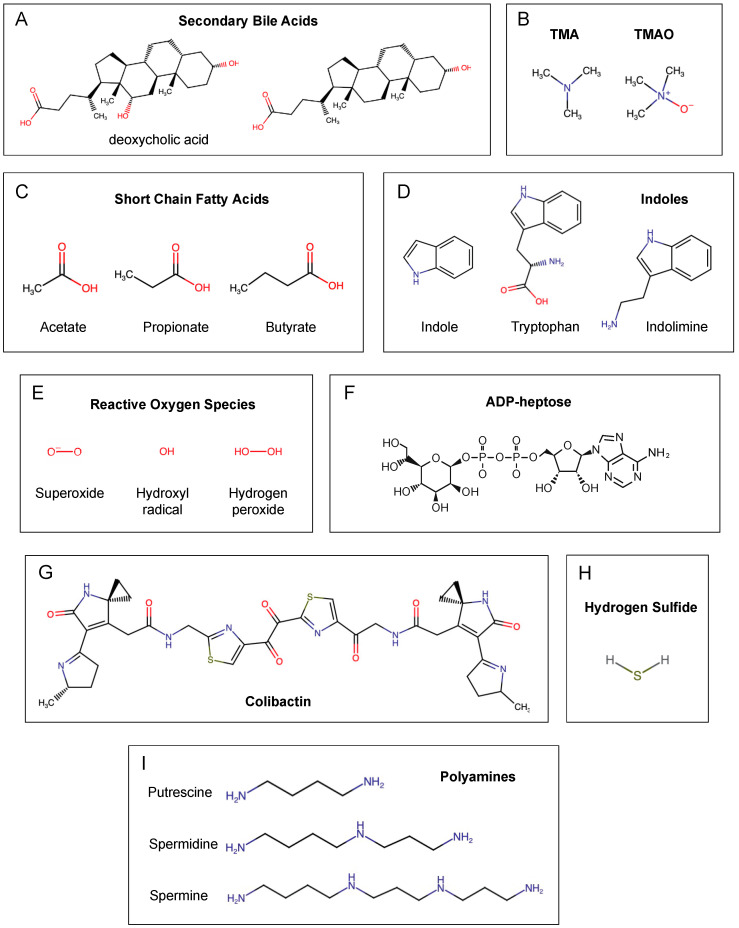
The chemical structures of the main bacterial metabolites associated with colorectal cancer: secondary bile acids (**A**); trimethylamine and trimethylamine-N-oxide (**B**); short-chain fatty acids (**C**); indoles (**D**); reactive oxygen species (**E**); ADP-heptose (**F**); colibactin (**G**); hydrogen sulfide (**H**); polyamines (**I**).

Although the majority of primary bile acids (~95%) are reabsorbed in the ileum and recycled in the liver, any remaining bile acids enter the colon, where they are susceptible to conversion into secondary bile acids by the bacterial microbiota (Figure 1A) [10,11,12,13]. Certain members of the microbiota have been reported to perform a wide range of enzymatic modifications. Secondary bile acids are often unconjugated as a result of the activity of bile salt hydrolases (BSH), which can deconjugate both primary and secondary bile acids [12]. BSH has been isolated from *B. fragilis*, *Bacteroides vulgatus*, *Clostridium perfringens*, *Listeria monocytognes* and the *Lactobacillus* and *Bifidobacterium* species [15]. Unconjugated bile acids can be converted by 7α-dehydroxylation, which generates deoxycholic acid (DCA) and lithocholic acid (LCA) from CA and CDCA, respectively [12,15]. The desulfation of bile acids can occur via sulfatases produced by *Clostridium*, *Peptococcus*, *Fusobacterium* and *Pseudomonas* [15]. Alternatively, the esterification of bile acids generates oligomers, which have been reported to occur in *Bacteroides*, *Eubacterium* and *Lactobacillus* [15]. Lastly, the oxidation and epimerization of bile acids can occur in the 3-, 7- or 12-hydroxy groups via bile acid hydroxysteroid dehydrogenases (HSDHs). Additionally, 3α- and 3β-HSDHs can be produced by a number of bacteria belonging to Bacillota phylum or by *Peptostreptococcus productus*, *C. perfringens* and *Eggerthella lenta* [15]. The *Clostridium*, *Eubacterium* and *Escherichia* species have been reported to perform complete 7-epimerization [15]. Various Bacillota members can produce 7β-HSDHs, while the *Clostridium*, *Eubacterium*, *Bacteroides* and *Escherichia* genera have members that express 7α-HSDHs. Furthermore, 12α- and 12β-HSDHs have also been detected in Bacillota members; however, no bacteria have been found to produce both [15].

### 2.2. Bile Acid Receptors

Primary and secondary bile acids are recognized by specific receptors, which can reveal more about their roles in inflammation and tumorigenesis. In particular, two receptors have been reported to be important in bile acid recognition. Firstly, the Farnesoid X Receptor (FXR) is a receptor that is expressed both in the liver and the intestinal tract. The FXR is activated most effectively by unconjugated primary and secondary bile acids, with varying affinities. FXR activation in the liver and intestines results in the inhibition of bile acid synthesis; however, the effect of FXR activation in the intestines is more pronounced [16]. Secondly, the G-protein-coupled bile acid receptor (TGR5) is a bile acid receptor that is ubiquitously expressed in humans [16]. The TGR5 recognizes both conjugated and unconjugated bile acids and is most efficiently activated by LCA and its conjugates, DCA and its conjugates, CDCA and its conjugates and GCA and its conjugates, with taurine conjugates always being the preferred ligands over glycine conjugates [16].

### 2.3. Role of Secondary Bile Acids in CRC

The dysregulation of bile acid metabolism has been associated with several diseases, including IBD and CRC. In IBD, secondary bile acids are less abundant in the feces of IBD patients and are at even lower concentrations in IBD patients with active disease flare-ups [17]. Furthermore, conjugated bile acids and sulfated bile acids are found in higher concentrations in IBD patients compared to healthy controls, suggesting that the microbiota converts these bile acids to a lesser degree [17]. Conversely, CRC risk is higher in patients who consume a high-fat diet, which results in increased levels of fecal secondary bile acids due to microbiota changes [18]. Therefore, secondary bile acids appear to have opposing disease associations in IBD compared to CRC as there is increased bile acid conversion in CRC but decreased conversion in IBD. However, both primary and secondary bile acid intestinal concentrations are largely dependent on enzymatic modifications by the microbiota and thus, microbiota composition, which can also change as a result of disease. Therefore, it is not always easy to determine whether bile acid alterations are the cause or consequence of disease.

Specific secondary bile acids play distinct roles in both diseases. For instance, in one study, mice were fed with DCA in their diet and they developed increased numbers of colorectal tumors and cancers [19]. More specifically, APC^min/+^ mice had a higher tumor burden when they were administered 0.2% DCA in their drinking water compared to the control mice [20]. This was associated with increased intestinal cell proliferation as a result of Wnt signaling. Additionally, DCA and, to an even larger extent, CDCA have been shown to induce apoptosis in a human colon adenocarcinoma cell line, which was suggested to be dependent on the induction of ROS formation [21]. In contrast, DCA and LCA seem to induce a protective effect during dextran sodium sulfate (DSS)-induced colitis in mice as the administration of these secondary bile acids per rectum results in an anti-inflammatory response and reduced colitis severity [22].

Another secondary bile acid that has been the center of research interest is ursodeoxycholic acid (UDCA). In a murine model of DSS-induced colitis, a physiological dose of UDCA protected against severe intestinal inflammation, likely as a result of the anti-inflammatory activity of UDCA [23]. However, this effect is likely dependent on the subsequent metabolism of UDCA into LCA by the intestinal microbiota since one analog of UDCA (6-MUDCA, which has shown a similar anti-inflammatory effect to that of UDCA in vitro but is unable to be metabolized by the microbiota) does not show this anti-inflammatory effect in mice [23]. In contrast, the administration of LCA protects against DSS-induced inflammation in mice to a similar extent as UDCA, indicating that UDCA itself does not drive the anti-inflammatory effect in the intestine but rather the downstream secondary metabolite LCA [23]. High-dose UDCA is currently applied for the clinical treatment of primary sclerosing cholangitis (PSC), while UDCA has also been implicated in the prevention of CRC initiation [24]. Since around 70% of PSC patients also have IBD, it is thought that UDCA administration could have a preventive effect on the development of CRC in these patients [24]. As a result, high-dose UDCA is thought to potentially play a role in preventing tumor development, specifically in UC patients who also have PSC [24]. However, when a high dose of UDCA was included in the diet of UC patients with PSC, the patients developed significantly more colorectal neoplasia in comparison to the placebo control group, showing that high-dose UDCA administration could in fact exacerbate CRC pathogenesis [24].

### 2.4. Impact of Bile Acid Receptor Stimulation on CRC Development

Various studies have examined the role of bile acid receptor stimulation to further understand its impact on inflammation and tumorigenesis. For instance, TGR5 stimulation in vivo and in vitro inhibits NF-κB signaling as result of lipopolysaccharide stimulation [25]. In a mouse model of colitis-associated colorectal cancer, the colons of treated mice showed a decreased expression of the FXR, while the TGR5 had increased expression levels [26]. This was further associated with a decrease in fecal secondary bile acids. The role of decreased FXR signaling in CRC has been further highlighted by T-βMCA, a mouse-specific primary bile acid that inhibits the FXR and, as a result, increases tumor development in APC^min/+^ mice [27]. Examinations have revealed FXR-dependent increases in the proliferation and induction of DNA damage in intestinal stem cells, which could be inhibited by the synthetic FXR agonist FexD [27]. The role of the FXR in immune regulation has also been described as the deletion of the FXR in dendritic cells results in an increase in regulatory T cells in mice [28]. The complex interplay between bile acids in the gut and liver was further exemplified by a recent study in which the FXR was specifically knocked out in the liver or intestinal epithelium of mice [29]. The results showed that liver-specific FXR deletion had the biggest effect on colonic gene expression and increased in the thickness of the intestinal mucus layer [29].

### 2.5. Summary

It is obvious that secondary bile acids can play an important role in the development and progression of CRC. However, additional rigorous research regarding this topic is needed to fully elucidate the complex interplay between bile acids, the microbiota and CRC. Several aspects of this interplay are particularly challenging. For instance, specific (secondary) bile acids administered to mice to determine their effect on CRC development can be metabolized by bacteria in the intestines, which may obscure the results. Therefore, it is key to perform experiments where all aspects, including the microbiota, are controlled as much as possible. Lastly, in vitro mechanistic experiments using human cell lines or organoids could further prove helpful in elucidating the effects of individual bile acids or receptors on cellular processes, which could eventually be translated into identifying their roles in CRC. This could also provide valuable human-specific insights as the bile acids present in mice are different from those in humans, as are the ligand specificities and signaling pathways of bile acid receptors.

## 3. Trimethylamine-N-Oxide (TMAO)

The diet-derived quaternary ammonium cations L-carnitine, choline and betaine can be metabolized by the intestinal microbiota into trimethylamine (TMA), which can subsequently be released into the gut lumen [30,31]. From there, TMA can enter the bloodstream and be converted into trimethylamine-N-oxide (TMAO) in the human liver by hepatic flavin monooxygenases (FMOs), primarily FMO3 and, to a lesser degree, FMO1 [30,31] (Figure 1B). Alternatively, TMAO can also be found in seafood and fish; thus, it does not require the microbiota or liver to produce or convert it [30]. The main source of L-carnitine is meat, while the main source of choline is eggs [30]. In addition, L-carnitine can also be synthesized endogenously in human cells from lysine and methionine, but this only accounts for ~25% of the L-carnitine pool [30,32]. Betaine is primarily derived from spinach, wheat germ and wheat bran [30]. Serum TMAO concentrations are positively correlated with several diseases, such as cardiovascular diseases (CVDs) and obesity [33,34]. Moreover, serum TMAO concentrations are also positively correlated with CRC [33,34,35,36].

### 3.1. Influence of the Microbiota on TMA and TMAO Levels

A study comparing the effects of various diets on the microbiota and TMAO plasma and urinary concentrations indicated that fish was the primary contributor to plasma TMAO and, therefore, was microbiota-independent [37]. Microbiota-dependent effects were observed following the consumption of eggs and beef via L-carnitine and choline, which contributed to increased TMAO plasma concentrations, while fruit consumption resulted in reduced TMAO concentrations [37]. Vegetarians have lower plasma and urine TMAO concentrations compared to omnivores [38]. One study reported that people with high levels of urinary TMAO appeared to have relatively more Bacillota but a lower abundance of Bacteroidota, although the sample size of the study was relatively low [37]. In agreement with this, bacterial species from the Bacteroidota phylum have been reported to be incapable of synthesizing TMA [39]. Using the oral carnitine challenge test, which was developed to examine microbiota-mediated increases in TMAO concentrations after the consumption of L-carnitine, it has been shown that high TMAO producers have a significantly different microbiota composition compared to low TMAO producers [38].

A different study found that all examined microbiotas were predicted to be able to synthesize TMA, based on genes found for choline metabolism (*cutC*) or L-carnitine metabolism (*cntA*) [40]. The *cutC* pathway was widely conserved in all microbiotas, while only 26% of the microbiotas contained the *cntA* pathway; both pathways were present at low abundances [40]. Additionally, the sequencing depth may be insufficient and other pathways could potentially be involved in their metabolism; thus, L-carnitine metabolism could be even more widespread. Indeed, a recent publication revealed that TMA-synthesizing pathways are more complex as bacteria can also harbor genes that produce the intermediate metabolite γ-butyrobetaine, which requires the cross-feeding of intermediary TMA metabolites to ultimately generate TMA [41].

### 3.2. Role of TMA and TMAO in CRC

In addition to the positive correlation between serum TMAO concentrations and CRC [33,34,35,36], serum TMAO concentrations are further associated with the abundance of *E. coli* and other members of the *Enterobacteriaceae* family in obese CRC patients [34]. However, serum TMAO concentrations do not predict the development of CRC in Finnish men, although the concentrations of serum choline do significantly correlate [42]. Serum TMAO concentrations may also have value as a predictor of chemotherapy response in CRC patients as high serum TMAO concentrations have been shown to lead to shorter disease-free survival times [36].

Despite the clear link between TMAO concentrations and CRC, the mechanisms are still very much under debate. One way in which TMAO may contribute to the development of CRC is via the induction of inflammation. At least in endothelial cells and smooth muscle cells, TMAO administration leads to an NF-κB-mediated pro-inflammatory response [43]. While TMA administration has a similar effect on NF-κB activation in these cells, TMA seems less relevant in non-intestinal models as their physiological concentrations are much lower compared to their TMAO concentrations [43]. Another study suggested that TMAO-induced endothelial inflammation was dependent on mitochondrial ROS-mediated NLRP3 activation [44]. The trace amine-associated receptor (TAAR) 5 is a receptor for TMA but not for TMAO [45,46,47]. Since it is an epithelial olfactory GPCR, the relevance of TAAR5 in the intestine has yet to be determined [46]. Alternatively, a potential receptor for TMAO could be protein kinase R-like endoplasmic reticulum kinase (PERK), which has been found in hepatic cells [48]. The activation of PERK could subsequently lead to NLRP3 and NF-κB activation [48]. In CRC cells specifically, TMAO has been shown to promote proliferation and, potentially, angiogenesis via the upregulation of vascular endothelial growth factor A [49].

### 3.3. Conclusions

While TMAO often takes the main stage, the question remains whether TMAO or TMA is the driving factor for CRC specifically. TMAO requires conversion in the liver first, while TMA is directly synthesized near the colorectal epithelium. A recent paper that looked at the effect of TMA on two colorectal epithelial cells lines (HT29 and HCT116) showed that TMA induced cell death and reduced proliferation in a dose-dependent manner [50]. Furthermore, the authors showed that TMA induced genotoxicity, which could further play a role in CRC development, although the mechanisms of this remain unknown [50]. Thus, although TMAO concentrations are correlated with CRC, they could just be a proxy for TMA concentrations in the intestine. Regardless, much more mechanistic research is required to fully understand the impacts and roles of TMA and TMAO in the pathogenesis of CRC.

## 4. Short-Chain Fatty Acids

In recent years, short-chain fatty acids (SCFAs) have been the most studied bacterial metabolites with regard to intestinal health. SCFAs are fatty acids with five or less carbon atoms and are the products of the fermentation of dietary fibers by the intestinal microbiota (Figure 1C) [7,12,51]. In particular, three SCFAs have been widely reported to contribute to colonic health: acetate, propionate and butyrate.

### 4.1. SCFA Production by Members of the Intestinal Microbiota

Acetate is the most generic and abundant SCFA in the colon, with concentrations about three times higher than those of butyrate and propionate, which is likely due to the fact that most intestinal bacteria are able to produce acetate [7,12]. However, butyrate has sparked the most interest with regard to its impact on the intestinal tract. While butyrate-producing bacteria are found across multiple intestinal phyla, the majority have been reported in the Clostridiales order within the Bacillota phylum, although not all members are able to produce butyrate [52]. Two of the most abundant butyrate producers within this order are *Faecalibacterium prausnitzii* and *Eubacterium rectale*, which together constitute around 12–14% of the total intestinal microbiota [52]. An extensive list of butyrate-producing bacteria is presented in [52]. The absence of aerobic bacteria, such as *Salmonella* and *E. coli*, is notable and could be explained by the fact that butyrate is utilized by the intestinal epithelium as its primary energy source and consumes O_2_ in the process [53,54]. A number of butyrate-producing bacteria have also been linked to the pathogenesis of CRC. For instance, most bacteria from the *Fusobacteriaceae* family have the butyrate synthesis pathway and are confirmed butyrate producers [55]. Similarly, the *Porphyromonadaceae* spp. contains several predicted and confirmed butyrate-producing strains. Lastly, as previously mentioned, many members of the *Clostridiaceae* family can produce butyrate [55]. In CRC patients, it has been reported that there is a decrease in butyrate-producing bacteria, such as *Faecalibacterium* spp. and *Roseburia* spp., compared to healthy individuals [56].

Propionate is also produced by a wide variety of bacteria, many of which are members of the Bacteroidota phylum and the Negativicutes class from the Baccilota phylum [7]. In addition, a number of individual bacterial species have been shown to produce propionate, including *Bacteroides thetaiotaomicron*, *Anaerostipes rhamnosivorans*, *E. coli*, *Roseburia inulinivorans*, *Blautia obeum*, *Salmonella* spp., *Listeria* spp., *Lactobacillus buchneri*, *Clostridium sphenoides*, *Eubacterium halli, Bifidobacterium* spp. and *Limosilactobacillus reuteri* [57,58,59].

### 4.2. SCFA Receptors

SCFAs are recognized by several different G-protein coupled receptors (GPCRs), including FFAR2 (GPR43), FFAR3 (GPR41) and GPR109a [60]. Most research so far has focused on FFAR2, which recognizes propionate and acetate but has a lower affinity with butyrate [61,62,63]. In contrast, FFAR3 has a preference for propionate and butyrate but barely responds to acetate [61,62,63]. Lastly, GPR109A responds to nicotinic acid (niacin) and β-hydroxybutyrate but can also sense butyrate and, to a lesser extent, propionate and acetate [60,64]. However, from its ligands, only butyrate is found in high enough concentrations to activate GPR109A in the colonic epithelium, which is one of the cell types with the highest expression levels [60]. The expression pattern of FFAR3 differs from that of FFAR2 as the expression of FFAR3 is higher in epithelial cells compared to immune cells, whereas FFAR3 is expressed in both epithelial and immune cells [60,65].

### 4.3. SCFA-Mediated Inhibition of Histone Deacetylases

One way in which SCFAs are reported to contribute to maintaining a healthy intestinal milieu is the inhibition of histone deacetylases (HDACs) [7,12]. HDACs are enzymes that deacetylate histones (the proteins that condense genomic DNA), thereby influencing the expression of genes [7]. Deacetylation leads to more condensed DNA packaging and, therefore, altered expression. In particular, butyrate and propionate are potent HDAC inhibitors [7,66,67]. While butyrate and propionate are believed to be able to directly inhibit HDACs, HDAC inhibition can also be dependent on SCFA receptors. For example, mice in which FFAR2 has been inactivated are more vulnerable to inflammatory diseases, such as colitis and arthritis [68]. This is in part explained by the FFAR2-dependent HDAC inhibition that leads to altered numbers of regulatory T cells in response to propionate [66]. Furthermore, the increase in Caco-2 cell migration and polarization as result of propionate stimulation is dependent on both FFAR2 and HDAC inhibition [69].

### 4.4. SCFAs As Immune Modulators

SCFAs have been extensively studied for their potential as immune modulators as they influence T cell differentiation and cytokine production through mechanisms that can involve both SCFA receptor activation and HDAC inhibition. For instance, multiple lines of research have shown that butyrate and propionate can induce the differentiation of regulatory T cells (Tregs) in colonic mucosa [66,67,70]. These studies have also revealed the role of the FFAR2-dependent inhibition of HDACs, either in Tregs directly or in dendritic cells, resulting in the Treg-dependent dampening of pro-inflammatory responses. Similarly, butyrate promotes IL-22 production via CD4^+^ T cells and innate lymphoid cells (ILCs), resulting in intestinal homeostasis, which is dependent on FFAR3 and the inhibition of HDACs but not on FFAR2 [71]. In contrast, a different study showed that the T cell differentiation induced by SCFAs was independent from both FFAR3 and FFAR2 and was solely dependent on the inhibition of HDACs [72]. Additionally, macrophages present in mucosa can be affected by SCFA-mediated HDAC inhibition, resulting in polarization toward a less inflammatory phenotype [73]. Finally, butyrate-mediated HDAC inhibition promotes CD8^+^ T cell function by upregulating IFNy production [74].

### 4.5. Role of SCFAs in CRC Pathogenesis

As described above, the presence of SCFAs in the intestinal tract generally leads to a less inflammatory intestinal environment and can contribute to a reduction in CRC development, which has been reported to occur via a variety of mechanisms [12]. In addition, butyrate-promoted CD8+ T cells have anti-tumorigenic properties and, therefore, provide a more direct mechanism by which SFCA-mediated immune modulation can contribute to CRC prevention [74,75]. FFAR2 has also been implicated in CRC. For instance, various studies have shown that the decreased expression of FFAR2 in CRC tissues and various colorectal cancer cell lines, which may indicate the role of FFAR2 in disease development or progression [76,77]. When FFAR2 expression is restored in HCT8 cells, the cells become more sensitive to apoptosis by butyrate and propionate and proliferate less as result of propionate treatment [77]. In contrast, when FFAR2 expression is silenced, the cells proliferate more in response to the supernatant obtained from the butyrate-producing *C. butyricum* [76]. FFAR2 expression and activation are also critical for preventing CRC development in mouse dendritic cells [78]. Mice with FFAR2 deletion are vulnerable to AOM/DSS-induced CRC, while mice with FFAR3 deletion are not [79].

SCFAs not only impact immune cells but also influence other cell types, such as those in the intestinal epithelium. Cancerous colonocytes, which shift their cellular metabolism to utilizing glucose instead of butyrate due to the Warburg effect, accumulate butyrate in their nuclei to promote HDAC inhibition [57]. This results in a decrease in proliferation. The inhibition of the Warburg effect also induces cell proliferation. Furthermore, butyrate and propionate induce apoptosis in a dose-dependent manner that is independent from the Warburg effect [57,77]. Similarly, the pro-apoptotic effect of butyrate on hepatic epithelial cells is dependent on both FFAR3 and HDAC inhibition [80].

However, there is also evidence that SCFAs, particularly butyrate, are not solely protective in CRC. For instance, a recent study claimed that butyrate production by bacteria in CRC tissues could further promote CRC development [81]. It was found that *Porphyromonas gingivalis* and *Porphyromonas asaccharolytica* were particularly enriched in CRC patients and secreted large amounts of butyrate into their environments [81]. This subsequently induced senescence in healthy epithelial cells and fibroblasts, which is strongly associated with cancer development [81,82]. In addition, *F. nucleatum* induces T helper 17 cells in mice that are pro-tumorigenic and FFAR2-dependent [83]. Although these are thought-provoking findings, conclusions on butyrate being the primary cause of tumor development in these tissues remain preliminary. However, these lines of research have indicated that the anti- or pro-tumor potential of SCFAs, specifically butyrate, could be dependent on the local concentrations and stages of tumor development [81].

## 5. Colibactin

The secondary metabolite colibactin is probably the CRC-promoting metabolite that is best understood at a mechanistic level [84]. Colibactin is a peptide polyketide that is produced by polyketide synthetase-producing (*pks*^+^) *E. coli* and some *Klebsiella pneumoniae* strains (Figure 1G). The *pks* gene is part of a gene island, termed the *pks* island, that expresses several genes required for the synthesis of colibactin [84]. Colibactin is considered a genotoxic metabolite. The structure of colibactin allows for its reactivity with DNA as two cyclopropane electrophiles are responsible for DNA alkylation [84,85]. *Pks^+^ E. coli* alkylates adenine residues within DNA, thereby inducing colibactin-DNA adducts, which are covalent DNA modifications, as shown in HeLa cells and in vivo colonic epithelial cells in mice [86]. Additionally, the interstrand crosslinking of DNA is induced in cells that are stimulated by colibactin-producing bacteria, which ultimately results in single- and double-stranded DNA breaks [86,87].

The extended exposure of colorectal organoids to *pks^+^ E. coli* has revealed that colibactin induces a specific mutagenic signature, which resembles the mutagenic signature seen in the tumor tissues of a subset of CRC patients colonized with *pks^+^ E. coli* [88,89]. This strongly suggests that patients colonized with colibactin-producing *E. coli* have a mutational burden that is strongly driven by this genotoxin. Colibactin-producing *E. coli* has been found to be enriched in CRC patients as ~97% of CRC samples in one study contained detectable *pks^+^ E. coli* [90]. Additionally, familial adenomatous polyposis (FAP) patients have biofilms primarily consisting of *pks^+^ E. coli* co-colonized with *B. fragilis*, suggesting the potential role of colibactin in the pathogenesis of FAP [91]. In addition to CRC, *pks^+^ E. coli* is also highly abundant in IBD patients, which could be a reason for the increased chance of developing CAC among these patients [92]. Altogether, colibactin and the bacteria that synthesize this genotoxin have been reported to contribute to CRC development.

## 6. Indoles

Several amino acid-derived metabolites have been reported to play a role in CRC development, particularly metabolites that are byproducts of tryptophan metabolism (Figure 1D). Diet-derived tryptophan is mostly metabolized by the host’s kynurenine (Kyn) pathway into a range of metabolites, including oxidized nicotinamide adenine dinucleotide (NAD+) [93]. Approximately 5% of dietary tryptophan is metabolized by the microbiota-based indolic pathway into various types of indoles [93]. Lastly, a small fraction of consumed tryptophan is converted to serotonin (5-hydroxytryptamine) and tryptamine, mostly via metabolism in the intestinal epithelium but also by the microbiota to a lesser extent [93].

### 6.1. Indole Production by the Intestinal Microbiota

The microbiota can produce indoles by metabolizing tryptophan using the tryptophanase (TnaA) enzyme. Alternatively, indoles can be synthesized via phenylalanine metabolism using phenylacetate dehydratase. Genetic variations in these enzymes lead to different indoles, such as indole-3-acetamide, indole-3-acetaldehyde and tryptamine [93]. Examples of bacteria that produce such indolic compounds include the *Peptostreptococcus, Clostridium, Bacteroides* and *Bifidobacterium* species [93]. Reductions in tryptophan to indole ratios in CRC patients are correlated with the reduced presence of *Asaccharobacter*, *Parabacteroides*, *Fusicatenibacter*, *Anaerofilum*, *Clostridium XIVb* and *Anaerostipes* [94]. Additionally, it has been shown that *Peptostreptococcus* is a key producer of indoleacrylic acid and indole-3-propionic acid [95].

### 6.2. Effect of Microbiota-Derived Indoles on Hosts

The effect of indoles on the host, immune system and CRC is generally considered beneficial [96]. One study found that a decrease in the indole to tryptophan ratio was observed in the feces of patients with CRC [94]. In mice, both indoleacrylic acid (IA) and indole-3-propionic acid (IPA) show anti-inflammatory effects on bone marrow-derived macrophages (BMDMs) but only IA exhibits the same effect in human PBMCs, indicating host species specificity [95]. Indoles further increase the transepithelial electrical resistance of cultured HCT-8 cells, while also having an anti-inflammatory effect after TNF stimulation [97]. Interestingly, indoles induce the upregulation of several cytokines, including IL-2, IL-4 and IL-10, and the downregulation of IL-8 [97]. In a mouse model of T cell-mediated colitis, IPA had an alleviating effect [98]. Indolic compounds have been further shown to affect epithelial barrier function and reduce inflammation via the upregulation of the IL10R1 receptor in intestinal epithelial cells [93,99]. Additionally, the administration of IPA to mice alleviates DSS-induced colitis [99]. These observed phenotypes could be (in part) caused by the effect of indoles on the activity of the aryl hydrocarbon receptor (AhR) as colonic epithelial cells stimulated with indoles lead to the upregulation of AhR target genes [100]. However, not all indole derivatives always show the same phenotypes; thus, the observed effects seem to be specific to certain indoles.

A recent study revealed an indolic metabolite termed indolimine, which is produced by the Gram-negative intestinal commensal *Morganella morganii* [101]. Indolimine was shown to directly induce DNA damage in purified DNA and cultured cells. Indolimine was furthermore shown to be involved in tumorigenesis in an AOM/DSS mouse model of inflammation-associated colorectal cancer as indolimine-producing *M. morganii* induced a higher tumor burden compared to *M. morganii* with an inactivated indolimine synthesis pathway [101]. Since *Morganella* is enriched in colon, rectum and stomach adenocarcinomas, it could be speculated that these bacteria and indolimine have similar effects in humans.

Altogether, while most tryptophan-derived indole metabolites appear to have anti-inflammatory and anti-tumor effects in the intestinal tract, several indole derivatives may also have detrimental effects on the host, with the tumor-promoting indolimine as a clear example.

## 7. Polyamines

Polyamines are small hydrocarbons that contain two or more amino groups and can be synthesized by both prokaryotes and eukaryotes. They are also present in our diets (Figure 1I) [102,103]. The most studied examples are the naturally occurring spermidine, spermine and putrescine [102]. Polyamines are positively charged and, therefore, can react with cellular molecules, such as proteins, DNA and RNA, thereby affecting several cellular functions, including gene expression and the functions of enzymes [102,104,105]. Additionally, spermine can protect DNA from oxidative damage induced by reactive oxygen species (ROS) by directly scavenging free radicals [106]. In the tumor microenvironment, including that of CRC, sustained high concentrations of polyamines are present [102,104]. This is believed to be caused by the dysregulation of the transcription factor c-Myc, which controls the rate-limiting enzymes required for polyamine production [102]. Polyamines have been implicated in CRC development as they are found to be elevated in proliferating and cancer cells [107].

### Role of Microbiota-Derived Polyamines in CRC

The intestinal microbiota has been reported to both utilize and synthesize various polyamines [108]. Therefore, polyamine contents in the intestinal tract can be positively or negatively influenced by the microbiota, depending on the microbial composition. Given the expected relevance of elevated polyamine concentrations in CRC, it could be argued that the microbiota could have a significant impact. The potentially beneficial roles of microbiota-derived polyamines were further exemplified in a study in which dietary polyamine consumption was measured in relation to CRC development [109]. Higher polyamine intake was correlated with a decrease in CRC risk, while the opposite was true for spermine [109]. However, to date, only one study has linked an individual microbiota member to tumorigenesis via the production of polyamines [110]: *F. nucleatum* was shown to produce high levels of putrescine after esophageal squamous cell carcinoma cellular invasion, which promoted the proliferation of tumor cells [110].

All in all, polyamines appear to play a role in CRC, and cancers in general, where an increase in polyamines in tissues is associated with disease. While the microbiota is both an active producer and utilizer of intestinal polyamines and can significantly influence tumor polyamine levels, additional data on the effect of the microbiota on polyamine levels in tissues and CRC need to be collected.

## 8. Hydrogen Sulfide

Hydrogen sulfide (H_2_S) (Figure 1H) is mostly produced by sulfate-reducing bacteria (SRB), which are dependent on inorganic sulfur in the intestinal tract [111,112]. SRB have been reported to be elevated in people with CRC [111,112]. SRB are most prominently present in the phylum Desulfobacterota [113]; however, several bacteria outside this phylum have been shown to harbor organic sulfur-metabolizing genes, such as *F. nucleatum* and *C. intestinale,* which are reliant on sulfur-containing amino acids [112]. Diets rich in processed meat, low-calorie drinks and alcohol result in an increased abundance of H_2_S-producing bacteria and an increased chance of developing CRC [114]. In CRC patients, the upregulation of endogenous and microbial H_2_S production is found in the intestinal tract, mainly with cysteine as a source [115]. Cysteine-reducing bacteria are an alternative producer of H_2_S that are more abundant in CRC samples compared to healthy controls. Additionally, sulfate-reducing bacteria are more abundant in CRC patients compared to healthy controls [113].

### CRC-Promoting and CRC-Inhibiting Functions of H_2_S

H_2_S can function as a scavenger of oxygen and reactive oxygen species, although it is debated as to whether this is relevant due to its low concentrations in the intestinal epithelium [116]. Another function of H_2_S is the persulfidation of proteins, which can occur on cysteine residues [116]. This has been shown to occur directly on NF-κB, where it mediates anti-apoptotic effects [116,117]. Elevated H_2_S levels in CRC can regulate cell invasion, while the inhibition of H_2_S can diminish cancer cell growth [116]. H_2_S has also been reported to be associated with DNA damage, epithelial cytotoxicity and the disruption of the mucus layer [111,118,119,120]. Inhibiting endogenous H_2_S production results in the reduced migration and angiogenesis of colorectal cancer cells [121].

However, conflicting reports exist on the effect of H_2_S on the development of CRC as it has also been shown to induce the protective autophagy pathway [118]. Additionally, increasing endogenous H_2_S production leads to inhibited colorectal cancer cell proliferation and migration [122]. Lastly, the nonsteroidal anti-inflammatory drug naproxen, which contains an H_2_S-releasing moiety, has been reported to inhibit the growth of HT-29 tumor cells [123].

In conclusion, H_2_S has been reported to have both CRC-inducing and CRC-protective effects. This could be explained by a bell-shaped dose–effect curve, with an ‘optimal’ H_2_S concentration that inhibits CRC progression while low or high concentrations of H_2_S promote CRC. Although multiple bacterial species are associated with the production of H_2_S and its likely involvement in CRC development, a direct causal role has not yet been identified.

## 9. Reactive Oxygen Species (ROS)

Reactive oxygen species (ROS) are reactive chemicals, including the superoxide radical (O_2_^•−^), the hydroxyl radical (OH^•^) and hydrogen peroxide (H_2_O_2_) (Figure 1H) [124,125]. ROS are well known to be produced by human cells, usually neutrophils, in an attempt to clear bacterial infections. The mechanism of action through which ROS kill bacteria is oxidization, which damages DNA, proteins and lipids [124,125]. However, some bacteria can also produce ROS via aerobic respiration [125].

### ROS Release by Bacteria

Thus far, only a very small number of known ROS- and/or RNS-releasing bacteria have been identified. *E. faecalis* is able to produce extracellular superoxide radicals, hydroxyl radicals and hydrogen peroxide in vivo in the intestines of rats [126,127]. *E. faecalis*-releases superoxide but not hydrogen peroxide and has been shown to promote CIN in cultured A_L_N cells (a hamster cell line containing human chromosome 11) [128]. That study also showed that superoxide produced by *E. faecalis* could induce COX-2 activation in macrophages, which in turn led to CIN in neighboring cells through diffusible factors [128]. Another bacterial species that has been shown to produce superoxide is *H. pylori*, which could play a role in the development of gastric cancer [129]. It remains to be seen whether more unidentified bacteria can produce and actively secrete ROS, thereby affecting host cells and driving tumor formation.

## 10. ADP-Heptose

ADP-heptose is a relatively recently discovered bacterial metabolite with inflammatory potential (Figure 1F) [130,131]. This metabolite is produced in various Gram-negative bacteria as part of the heptose biosynthesis pathway, which forms heptose residues that are incorporated in the core of lipopolysaccharide, a major component of Gram-negative outer membranes [132]. A limited number of Gram-positive bacteria can also produce ADP-heptose, such as *Streptomyces fimbriatus* and *Streptomyces hygroscopicus* [132]. In host cells, ADP-heptose is recognized by the cytosolic alpha-kinase 1 (ALPK1) receptor, which initiates a signaling cascade via TIFA and TRAF2/6 to activate the pro-inflammatory transcription factor NF-κB [130]. In addition to pro-inflammatory signaling, ALPK1 activation and mutations have also been linked to the development of tumors [133,134,135,136,137]. For example, *F. nucleatum* abundance is correlated with ALPK1 expression in CRC tissues, which is further correlated with lower survival rates in these patients [133].

While the number of known bacteria that release ADP-heptose into its environment or directly into host cells is still rather limited, some are associated with the development of colorectal cancer. For instance, *Helicobacter pylori* injects ADP-heptose into host cells via its Type IV secretion system, which induces DNA damage as a result of R-loop formation (co-transcriptional RNA/DNA hybrids) [138,139]. It has been hypothesized that these effects could potentially contribute to gastric cancer development from chronic *H. pylori* infection in humans [138,139]. *Campylobacter jejuni* has been shown to activate ALPK1 by releasing ADP-heptose into its environment, which is subsequently taken up by host cells. While *C. jejuni* usually causes a self-limiting transient enteritis, several reports have linked *C. jejuni* infection to an increased risk of gastrointestinal cancer [140,141,142,143]. Whether ADP-heptose plays a role in this remains to be seen. *F. nucleatum*-dependent ALPK1 activation, which is likely caused by the release of ADP-heptose into its environment, induces the increased adhesion of CRC cells to endothelial cells in an ICAM-1-dependent manner, which may potentially contribute to increased cancer metastasis [133].

## 11. Concluding Remarks

**Figure 2:**
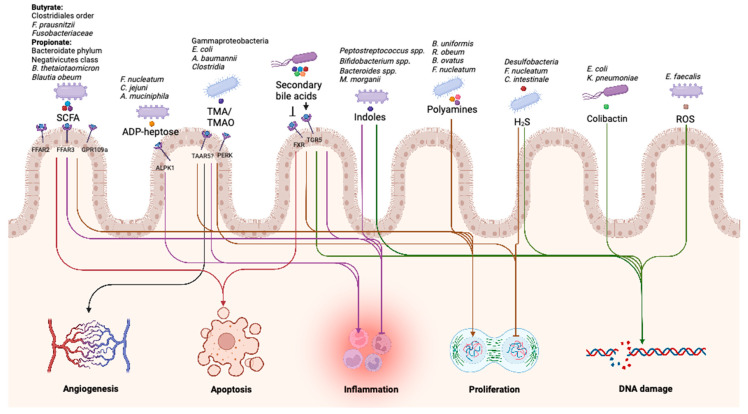
An overview of the main bacteria, their metabolites, (potential) receptors and the ultimate effects of their metabolites on the hallmarks of colorectal cancer. Created with BioRender.com.

In conclusion, the microbiota produces a plethora of different metabolites that can positively or negatively contribute to the development of colorectal cancer (Figure 2). Among these metabolites, secondary bile acids, colibactin, TMA/TMAO and butyrate have been found to play an important role in the development of CRC. The other metabolites discussed in this review either have a weaker association with CRC or are less well understood mechanistically; nonetheless, they may also be important players in the development of CRC.

One aspect that hampers this field of research is the complexity of bacterial metabolites and their host receptors. For instance, while SCFAs are often described together, butyrate can have completely different effects on host cells than propionate or acetate and their receptors are variably expressed in host cells and have different affinities for individual SCFAs. Similarly, the vast array of primary, secondary and tertiary bile acids in the intestinal tract, which are strongly dependent on microbiota composition, are at the heart of a complex interplay involving the microbiota, host metabolism and the regulation of bile acid uptake and production [16,26,144]. Lastly, while indoles generally seem to have a positive impact on health, there are indolimines that can promote cancer development [94,95,96,97,98,101]. Metabolites also often have dose-dependent effects on the host, adding another layer of complexity [12,57,116,145]. Therefore, deciphering the precise roles of individual bacterial metabolites in vivo continues to be a challenge.

While there is still a lot of progress to be made to delineate the exact effects of individual metabolites on the host and CRC development, research conducted over the last decade has clearly highlighted the importance of microbiota-derived metabolites in the pathogenesis of CRC. These findings will undoubtedly lead to novel diagnostic and therapeutic options in the future.

## Data Availability

Not applicable.
